# Effectiveness and Usability of Digital Tools to Support Dietary Self-Management of Gestational Diabetes Mellitus: A Systematic Review

**DOI:** 10.3390/nu14010010

**Published:** 2021-12-21

**Authors:** Nurudeen Adesina, Huseyin Dogan, Sue Green, Fotini Tsofliou

**Affiliations:** 1 Department of Rehabilitation and Sport Sciences, Faculty of Health & Social Sciences, Bournemouth University, Bournemouth BH8 8GP, UK; nadesina@bournemouth.ac.uk; 2 Centre for Midwifery, Maternal & Perinatal Health, Faculty of Health & Social Sciences, Bournemouth University, Bournemouth BH8 8GP, UK; 3 Department of Computing and Informatics, Faculty of Science and Technology, Bournemouth University, Poole BH12 5BB, UK; hdogan@bournemouth.ac.uk; 4 Department of Nursing Science, Faculty of Health and Social Sciences, Bournemouth University, Bournemouth BH8 8GP, UK; sgreen@bournemouth.ac.uk

**Keywords:** gestational diabetes mellitus, lifestyle, dietary management, digital tool, smartphone apps

## Abstract

Advice on dietary intake is an essential first line intervention for the management of gestational diabetes mellitus (GDM). Digital tools such as web-based and smartphone apps have been suggested to provide a novel way of providing information on diet for optimal glucose regulation in women with GDM. This systematic review explores the effectiveness and usability of digital tools designed to support dietary self-management of GDM. A systematic search of Medline, Embase, Cumulative Index to Nursing and Allied Health Literature (CINAHL), Cochrane Library, and Scopus using key search terms identified 1476 papers reporting research studies, of which 16 met the specified inclusion criteria. The quality of the included studies was assessed using the ErasmusAGE Quality Score or the Mixed Methods Appraisal Tool (MMAT) version 2018. The findings show that the adoption of digital tools may be an effective approach to support self-management relating to healthy diet, health behaviour, and adherence to therapy in women with GDM as a usable intervention. However, there is a lack of evidence concerning the effectiveness of tools to support the dietary management of GDM. Consideration for ethnic specific dietary advice and evidence-based frameworks in the development of effective digital tools for dietary management of GDM should be considered as these aspects have been limited in the studies reviewed.

## 1. Introduction

Pregnant women with gestational diabetes mellitus (GDM) are at increased risk of adverse maternal and foetal outcomes [1,2]. Globally, GDM prevalence ranges between 1% and 28%, depending on population setting and diagnostic criteria used [3]. In the United Kingdom, the condition is diagnosed in 16 out of every 100 pregnant women [4]. Asian and Black minority people have the highest burden of GDM with a prevalence of 46% and 43%, respectively, in the UK [4,5]. Management of GDM is aimed at achieving optimal glucose regulation through dietary and other lifestyle modifications, improvement of psychosocial care, and prevention of complications [6]. Consumption of food with low glycaemic index (GI) improves maternal glucose control, which consequently results in the reduction in neonatal glycaemic loads [7]. The ability of dietary interventions to be adapted and tailored to the needs of targeted populations make it an essential first line intervention for the lifestyle management of GDM [3]. Emerging evidence suggests that a high quality diet such as the Mediterranean diet (MD)—high consumption of fruits, vegetables, nuts, and legumes, extra virgin olive oil as the principal fat source; moderate consumption of poultry, eggs, and dairy; only occasional consumption of red meat—can be acceptable in women of childbearing age who wish to optimise their health for the prevention of chronic diseases such as diabetes [8]. Furthermore, a randomised trial found that a Mediterranean-style diet could be beneficial in reducing gestational weight gain and incidence of GDM in high risk pregnant women [9]. Therefore, appropriate dietary advice can support women with GDM to achieve improved dietary and other self-management approaches to GDM [10].

Digital tools are information and communication technologies such as smartphone apps, patient portals, and many other Internet-based programs or software designed to access information [10,11]. Research has revealed that smartphone and web-based digital tools have the potential to facilitate positive self-management of all forms of diabetes including GDM [12,13]. Adoption of health-related digital tools in pregnancy is often associated with intention to manage diet, physical activity, and other self-management routines such as weight monitoring, glucose reading, and tracking [7]. Two recent studies on the effectiveness of smartphone and web-based technologies to support health care during pregnancy in high-income countries concluded that the dietary advice included in many digital tool technologies is not consistent with the existing evidence-based dietary guidelines and often contained general information not tailored to the specific dietary needs of many users [14,15].

Effective utilization of evidence-based dietary advice for pregnant women with GDM to support them to make well informed decisions for optimal glucose regulation calls for integrated collaboration between health researchers, professionals, and digital tool developers [15]. Exposure to misinformation on dietary and other lifestyle management can increase the risk of GDM-related complications such as caesarean section, higher need for induction of labour and preeclampsia in pregnant women as well as congenital defects in the affected babies [16]. There is a need for an all-encompassing assessment of dietary digital tools through the evaluation of the usability in terms of acceptability and feasibility as well as the effectiveness towards achieving better pregnancy outcomes in women with GDM [17]. The effectiveness of the dietary digital tools for self-management of GDM is the extent to which the tools improve pregnancy outcomes [18,19]. Acceptability is evaluated through user satisfaction, appreciation, and recommendation to others for dietary self-management [14]. The feasibility of digital tools is assessed in terms of actual use, intention to use, and perceived appropriateness [20]. To achieve better health outcomes in women with GDM, digital tools for dietary management and other lifestyle management should be effective, evidence-based, and found usable by the intended users [18]. However, there is limited evidence to inform the effectiveness and usability of existing smartphone and web-based digital tools to improve pregnancy outcomes in women with GDM through the adoption of a healthy diet, blood glucose monitoring, and other lifestyle practices [14]. Hence, this systematic review was conducted to explore the effectiveness and usability of existing digital tools to support dietary self-management in women with GDM.

## 2. Materials and Methods

### 2.1. Design

A systematic literature review was undertaken on published primary studies reporting digital tools to support self-management of GDM. Systematic review is high on the hierarchy of scientific research as it synthesises the results of relevant studies to yield less-biased combined knowledge [21]. The methodology adopted for the systematic review was in accordance with the University of York Centre for Reviews and Dissemination [22], Cochrane Handbook on Systematic Review, and the Preferred Reporting Items for Systematic Review and Meta-analysis (PRISMA) guidelines [23].

### 2.2. Search Strategy

We conducted a systematic review of studies on dietary digital tools to support lifestyle management of gestational diabetes mellitus. We searched the databases Medline (via the Web of Science), the Cochrane Central Register of Controlled Trials (CENTRAL), Embase, Cumulative Index to Nursing and Allied Health Literature (CINAHL), and Scopus using a combination of Medical Subject Headings (MeSH) and free text to cover the search terms. The search strategy is shown in Supplementary Materials. The key words were combined by the EBSCO host operator AND/OR. Supplementary Literature searches involved examining the reference lists of all relevant studies. The literature search was conducted between September 2020 and January 2021 according to a predefined protocol. The search was limited to human studies, reported in the English language, and no time restrictions were applied.

### 2.3. Inclusion Criteria

Original research studies reporting qualitative and quantitative studies of digital tools (web-based, telemedicine and smartphone app-technology) targeting dietary and lifestyle support for women with GDM were included. Inclusion criteria included studies in which:

#### Pregnant Women with a Diagnosis or History of GDM Participated

Digital tools focusing on dietary and/or lifestyle management of gestational diabetes mellitus were investigated;

Randomized controlled trials, pilot studies, prospective or retrospective cohort studies, survey and mix methods were used; and

Outcomes relating to digital tools for management of gestational diabetes mellitus were reported.

### 2.4. Exclusion Criteria

Exclusion criteria were studies in which:

Pregnant women with no diagnosis or history of GDM participated;

Digital tools that did not include dietary advice;

Review articles or opinion publications; and

Abstracts and unpublished studies were not included in this systematic review.

### 2.5. Data Extraction

The initial screening was performed by the first reviewer (NA) and included a review of all titles and/or abstracts compared to the eligibility criteria, and a second reviewer (FT) offered consensus to ensure no relevant study was erroneously excluded. Data were extracted by the first reviewer (NA) from studies that fulfilled the inclusion criteria, and a proportion of the extracted data (30%) was checked for accuracy by the second reviewer (FT). Conflicts were resolved by discussion, or arbitrated if necessary, with the third reviewer (SG).Similarly, if eligibility was unclear, this was discussed across the wider team (NA, FT, SG, and HD). The search was completed by checking the reference lists of the included articles for studies not found in the database search. Reference management software EndNote version 20 was used to combine the search results from the electronic databases and remove duplicated records. A standardised form adjusted for this study was used for data extraction. The information extracted included author, aim of the study, participants, study intervention, and key findings.

### 2.6. Quality Assessment

The ErasmusAGE quality assessment tool for systematic reviews was used to assess the quality of RCTs included in the review. The ErasmusAGE quality score is applicable to both interventional and observational studies [24,25]. The tool is composed of five items and each item is allocated 0, 1, or 2 points, thus summarises a total score between 0 and 10 points in which 10 points represent the highest quality [24]. The Mixed Methods Appraisal Tool (MMAT) version 2018 was used to evaluate the quality of the included mixed method, qualitative and quantitative research articles [25]. Quality assessment was conducted independently by NA for all included studies. FT undertook an independent check to ensure the accuracy of quality scores for all included papers. The findings from the two reviewers were compared and any contrasting items of quality scores were discussed with the other two reviewers (SG and HD).

## 3. Results

### 3.1. Study Selection

**Figure 1:**
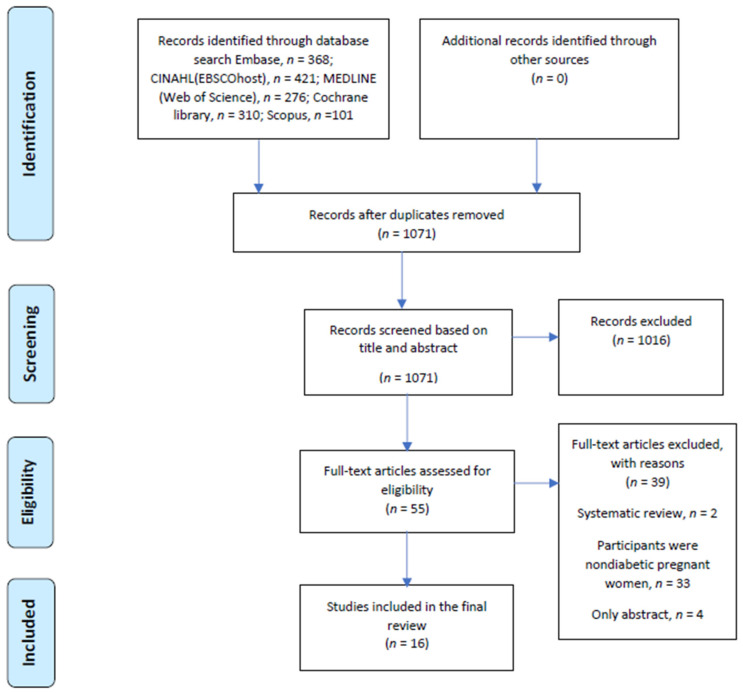
Preferred Reporting Items for Systematic Reviews and Meta-Analyses (PRISMA) flow diagram for the search strategy and study selection process.

The initial search identified 1476 titles and abstracts of which 1071 research studies remained after deduplication. After the review of the title and abstract, the full text of 55 articles that were potentially relevant was retrieved for further examination, and their references were manually screened to identify articles not included in the original search. However, the process yielded no additional articles. After reading the full articles, a total of 16 studies [26,27,28,29,30,31,32,33,34,35,36,37,38,39,40,41] met the inclusion criteria (Figure 1).

### 3.2. Study Quality Assessment

Twelve RCTs [27,28,29,30,31,34,35,37,38,39,40,41], two mixed method studies [33,36], and two qualitative studies [26,32] were included in this review. Of the RCTs included in the review, ten studies were graded as high quality because they scored 6 to 7 in the ErasmusAGE quality assessment score [27,29,30,31,34,35,37,38,40,41], and two studies [28,39] were graded moderate because they scored 5 out of the total score of 10 points (Table 1).

The remaining four studies [26,32,33,36] assessed using the Mixed Methods Appraisal Tool (MMAT) version 2018 were of moderate quality (Table 2).

### 3.3. Study Characteristics

The sixteen studies included in the review with a total of 2593 participants were published between 2009 and 2020. In total, nine studies (*n* = 1707) [28,30,31,34,37,38,39,40,41] focused on the effectiveness of digital tools to promote a healthy diet, monitoring of blood glucose, and other lifestyle practices. Three studies (*n* = 338) [29,32,35] evaluated acceptability of dietary digital tools in terms of user satisfaction, perception, and recommendation to others. Four studies (*n* = 548) [26,27,33,36] evaluated feasibility in terms of actual use, intention to use, and perceived appropriateness of the digital tools for the lifestyle management of GDM. Study locations were from three geographic regions including Europe (10/16) [29,31,32,35,36,37,38,39,40,41], Asia (4/16) [28,30,33,34], and Australia (2/16) [27,39]. Regarding specific digital tools, nine studies [26,28,30,31,32,33,34,36,41] investigated mobile apps while seven studies [27,29,35,37,38,39,40] were web-based dietary digital tools for managing GDM.

### 3.4. Findings

#### 3.4.1. Effectiveness of Digital Tools to Support Dietary Self-Management of GDM

The characteristics of nine studies [28,30,31,34,37,38,39,40,41] that reported on the effectiveness of digital tools in terms of promoting a healthy diet, monitoring blood glucose, and increasing physical exercise in women with GDM can be seen in Table 3. In five of the studies [27,31,34,39,40], women using the digital tool for dietary self-management had significantly lower glycaemic index, body mass index, gestational weight gain, and increased physical exercise compared with women in the control group. In addition to standard care, women recruited into the intervention group in Guo et al. [34] demonstrated higher levels of compliance (83.3 ± 12.5% vs. 70.4 ± 10.1%, t = −6.293, df = 122, *p* < 0.001), lower use of outpatient services (8.1 ± 1.3 vs. 11.2 ± 1.1, t = 14.285, df = 122, *p* < 0.001), lower glycosylated haemoglobin before delivery (4.7 ± 0.2 vs. 5.3 ± 0.3, t = 13.216, df = 122, *p* < 0.001), and lower weight gain (3.2 ± 0.8 vs. 4.8 ± 0.7, t = 11.851, df = 122 *p* < 0.001) than the control group. However, there were no statistically significant differences between the two groups in terms of pregnancy related complications such as macrosomia, preeclampsia, and perinatal complications. Caballero-Ruiz et al. [40] reported that the adoption of a web-based support system for dietary and insulin management in women with GDM reduced face-to-face visits to the hospital by 88.6% as well as GDM adverse outcomes. Nevertheless, no data on the effectiveness of the intervention on dietary habit and GDM outcomes was reported by Caballero-Ruiz et al. [40]. Furthermore, one study [39] reported that more women in the intervention group experienced weight loss (90.4% vs: 48.3%. *p* < 0.001) and were considered to have healthy weight postpartum (BMI = 18.5, 24.9 kg/m^2^ at 12 weeks postpartum (96.2% vs. 70.7% *p* < 0.001) than the control group. However, there was no significant difference in the infant weight at birth in both groups. In addition, the study was a non-blinded single centre trial of which the results may not be generalisable to other populations.

In a study by Miremberg et al. [30], participants in the intervention group received routine clinic-based GDM standard care with smartphone-based daily feedback apps while the control group only received standard care. The intervention group demonstrated improved compliance with dietary advice (84 ± 0.16% vs. 66 ± 0.28%, *p* < 0.001), glycaemic control (105.1 ± 8.6 mg/dL vs. 112.6 ± 7.4 mg/dL, *p* < 0.001), and communication with health care professionals, but no difference in overall pregnancy outcome in both groups. In contrast, Dalfra et al. [37] reported significant improvement in glucose monitoring and overall pregnancy outcomes among women with GDM using the telemedicine intervention. Borgen et al. [41] compared the effectiveness of the Pregnant+ app on the 2-h glucose level of the routine postpartum oral glucose tolerance test (OGTT) among women with gestational diabetes mellitus (GDM) in 238 women with GDM and found no significant different in the rate of change of glucose level between the intervention and the control group. Loss to follow-up and insufficient power of the study to perform subgroup analysis among participants from Somali and Pakistani ethnic minorities were given by Borgen et al. [41] as a potential reason for the ineffectiveness of the tools among the ethnic minorities. Nevertheless, the intervention group reported in the qualitative comments that the apps increased their dietary management of their condition more than in the control group. Evidence on the effectiveness of digital tools to support dietary management of GDM is still lacking; nevertheless, adoption of web-based and smartphone digital tools can significantly reduce hospital visits in women with GDM.

#### 3.4.2. Acceptability of Digital Tools to Support Dietary Self-Management of Gestational Diabetes Mellitus

Acceptability of digital tools for dietary management of GDM was assessed by user satisfaction, appreciation, and recommendation to others [42]. In total, three studies [29,32,35] reported on acceptability of the digital tools for dietary management of GDM (Table 4). Most of the participants in two studies [29,32] agreed that digital health was acceptable and feasible for lifestyle adjustment towards the effective management of gestational diabetes. Hirst and Mackillop [32] reported satisfaction and acceptability of smartphone-based digital health tools for dietary and weight management in over 80% of women with GDM. Smartphone GDM management intervention in the study by Rigla et al. [29] was observed to facilitate higher glucose monitoring compliance, modification of dietary habit by consuming food with low glycaemic index, and changes in gestational weight gain. However, the small sample size and absence of the control group in the study call for caution. The study by Given et al. [35] found that 89% of participants was satisfied with the digital tools and agreed to use them again as adjunct to standard clinical care for management of their hyperglycaemia. Although 83% of participants in the study by Hirst et al. [32] agreed that integrating digital tool intervention into the antenatal care pathway for lifestyle management of their condition was useful and increased their understanding on how GDM could be effectively managed through diet and other lifestyle approaches, concerns were raised about some featured information such as commercial advertisements and weather conditions in most of the tools that were not relevant to their needs.

#### 3.4.3. Feasibility of Digital Tools to Support Dietary Self-Management of Gestational Diabetes Mellitus

Feasibility of digital tools was assessed in terms of actual use, intention to use, and perceived appropriateness [20]. In total, four studies [26,27,33,36] examined the feasibility of digital tools to support dietary and other lifestyle management approaches for GDM (Table 5). Three of these [26,27,36] reported that the majority of the women in their studies learnt how to self-manage their GDM such as by measuring their blood glucose values, adjusting their diets, and physical activity by using an interactive and smartphone-based digital intervention. Participants in the study by Skar et al. [26] accepted that smartphone apps facilitate easily accessible dietary advice and overview of the glucose value with potential to improve the dietary management of GDM. However, the participants complained that dietary advice and the suggested baseline glucose limit in the apps were not always in agreement with the recommendations from their midwives. While evaluating the usability of a web-based dietary digital tool, Gian Francisco et al. [36] reported that the measure of usability of the dietary digital tool through the System Usability Scale (SUS) was found to be good (mean 70.9, 95% CI 67.1, 74.6), thus the tool has the potential to support self-management of GDM among pregnant women with the condition. Nevertheless, a key limitation in the study is the responder demographic characterized by fewer numbers of participants from individuals from lower socioeconomic backgrounds as well as minority ethnic groups. Therefore, further research to explore the uptake of digital tools for the dietary and lifestyle management of GDM among the minority ethnic groups and lower socioeconomic background is recommended [36]. Lack of reminders for blood glucose monitoring, diet control, and physical exercise were reported by Hewage et al. [33] as barriers to the management of GDM, however, the majority of the participants (174/215, 80.9% vs. 116/215, 53.9%) preferred to use the digital tools as additional support to dietary and other lifestyle advice received directly from the health care provider [33].

## 4. Discussion

There has been a rapid development in health information technology and health care related technology globally [43]. The consequent emergence of technology tools such as smartphone and digitally enabled interventions has attracted great interest in the use of these tools in public health and lifestyle medicine, both in research and practice [44]. Due to their cost effectiveness and promising potential to enhance health behaviour change [45], digital tools can be integrated into novel approaches for lifestyle improvement pertaining to population health [46]. The fundamental goal of digital health is to support lifestyle practices for effective prevention and management of health conditions [30,34]. Online and smartphone health apps have been found to have a positive impact on the prevention of disease through virtual consultation, online support groups, and web-based expert guidance on health conditions [45,47]. In addition to reducing unnecessary visits to health care services for health advice, smartphone and other Internet-based health platforms also facilitate better patient engagement, maintaining appointments and early reporting of any health signs of concern to health care practitioners [46]. Hence, health digital tools consolidate public interest for more affordable and easier access to their health needs [12,19].

Due to the success recorded by Internet-based and smartphone health advice, dietary digital tools have been suggested to provide novel information on the dietary needs for optimal glucose regulation among women with gestational diabetes mellitus [38,41]. Management of GDM is aimed at achieving optimal glucose regulation through dietary and other lifestyle modifications, improvement of psychosocial care, and prevention of complications [6]. Digital tools using web and smartphone-based technology have been developed to improve dietary habits, activity levels, and other lifestyle factors for better health outcomes [48]. Limited research has examined the effectiveness and usability of digital tools designed to support dietary self- management of GDM [49].

Based on findings of this review, digital tools to support lifestyle improvement relating to healthy diet, health behaviour, and adherence to therapy in women with GDM were found to be an acceptable and feasible intervention. However, there was no significant difference in pregnancy outcomes between the intervention and control groups. This resonates with previous literature reviews [14,50,51]. According to Badawy et al. [51], adopting digital tools has been found to be acceptable and feasible to facilitate healthy eating, physical activity, and weight management in adolescents. Rasekaba et al. [50] considered digital intervention facilitates healthy diet through easier access to dietary advice for self-management of health conditions in pregnancy, however, there are not sufficient data on the effectiveness of the tools on the prevention of pregnancy related complications in women with GDM. Few studies have explored dietary intervention targeting weight lost in women with GDM [52,53,54]. Effective weight management and return to healthy weight range was achieved by participants who adhered to online dietary advice to manage their gestational diabetes in one of the included studies [39]. These findings agreed with another review by Vickery et al. [55] that found that effective utilisation of mobile and web-based health intervention promoted healthy gestational weight gain in pregnancy.

Findings from Overdijkink et al. [14] while evaluating the usability and effectiveness of mobile health lifestyle intervention to support health care during pregnancy revealed that the tools are a feasible and acceptable intervention to increase intakes of vegetables and fruit as well as a reduction in gestational weight gain among pregnant women. However, further investigation into the effectiveness of the digital tools for lifestyle management of health conditions is recommended. Smartphone digital tools are feasible and acceptable among people with type 2 diabetes mellitus to enhance glycaemic control through adherence to nutrition guidelines and physical activity, nevertheless, research on the effectiveness and how the use of smartphone and web-based digital health intervention meet the specific needs of different subgroups of diabetes patients should be considered [56].

Most of the studies in this review [26,28,30,31,33,34,37,38,39,40,41] reported a low attrition with corresponding high retention rate. According to the Cochrane Handbook for Systematic Reviews of Interventions, studies with retention rates over 80% are classified as having low attrition [57]. Therefore, it can be assumed that the high feasibility and acceptability of digital tools for managing GDM identified in this review explain the high retention rate among users. Thus, smartphone and web-based digital tools could be effectively adopted by users for dietary management that will foster better maternal and foetal outcomes in women with GDM.

### Strengths and Limitations of the Study

Despite the limited evidence on the effectiveness, our systematic review found that smartphone and web-based dietary digital tools is a usable intervention in terms of feasibility and acceptability for lifestyle management of gestational diabetes. This present review adds to the body of knowledge and will help to guide future research on the development of a user-specific digital tool intervention for dietary management of gestational diabetes mellitus. However, the study has some limitations. Our study was limited to primary research published in English, which may have excluded relevant studies in other languages. None of the included study was conducted in Africa. Hence, our findings could not draw any conclusion regarding the adoption of dietary digital tools for managing gestational diabetes mellitus in low-income countries. This highlights the importance of conducting similar studies in a low socioeconomic society. Furthermore, we observed selection bias due to the lack of allocation concealment in all included RCTs. In addition, blinding of participants was only reported in one of the studies [41], thus indicating performance bias. The current systematic literature review did not include a meta-analysis due to the heterogeneous nature of the data and relatively small number of the included studies. There was no prospective registration of this systematic review conducted on PROSPERO, which is considered as one of the limitations of this study.

## 5. Conclusions

This review focused on the effectiveness and usability of digital tools on the dietary management of gestational diabetes mellitus. Based on our findings, digital tools to support lifestyle improvement relating to healthy diet, health behaviour, and adherence to therapy in women with GDM were found to be an acceptable and feasible intervention. However, there was a lack of evidence concerning the effectiveness of the tools to support dietary management of GDM. The results suggest consideration for user-specific dietary advice and an evidence-based framework in the development of effective digital tools for the dietary management of GDM.

## Figures and Tables

**Table 1:** Quality scores for included RCTs in the review using the ErasmusAGE quality assessment tool.

Author	Design	Size	Exposure	Outcome	Adjustment	Total	Quality
Borgen et al. [41]	2	2	1	2	1	8	High
Guo et al. [34]	2	2	0	2	0	6	High
Given et al. [35]	2	1	0	2	1	6	High
Caballero-Ruiz et al. [40]	2	1	0	2	1	7	High
Dalfrà et al. [37]	2	2	0	2	0	6	High
Miremberg et al. [30]	2	2	0	2	0	6	High
Carolan-Olah and Sayakhot [39]	2	2	0	1	0	5	Moderate
Rigla et al. [29]	2	2	0	2	0	6	High
Kennelly et al. [31]	2	2	0	2	0	6	High
Carral et al. [38]	2	2	0	2	0	6	High
Sayakhot et al. [27]	2	2	0	2	0	6	High
Roozbahani et al. [28]	2	1	0	2	0	5	Moderate

**Table 2:** Summary of quality assessment for included qualitative, quantitative, and mixed method studies using the Mixed Methods Appraisal Tool (MMAT) version 2018.

Author	Study Design			Score
	Qualitative	Quantitative	MM	
Hewage et al. [33]	**	**	**	50%
Gianfrancesco et al. [36]	**	**	**	50%
Hirst et al. [32]		**		50%
Skar et al. [26]	**			50%

Scoring descriptors for MMAT quality assessment: ** (50%).

**Table 3:** Overview of randomised controlled trials reporting on the effectiveness of digital tools to support dietary self-management of GDM.

Author (Country)	Aim of the Study	Participants, Setting	Study Intervention	Key Findings
Borgen et al. [41] (Norway)	To assess the effectiveness of a “pregnancy+ “app on Glu levels	238 women, 5 diabetes clinics	Intervention (*N* = 115): pregnancy+ app and usual care Control (*N* = 123): usual care	NS difference in Glu levels [6.7 mmol/L (95% CI 6.2 to 7.1) vs. 6.0 mmol/L (95% CI 5.6 to 6.3)] intervention vs. control
Caballero-Ruiz et al. [40] (Spain)	To evaluate the effectiveness of a web-based support system (Sinedie) on diabetes clinic visits	90 pregnant women with GDM, diabetes clinic	Intervention (*N* = 60): Web-based support system and standard care Control (*N* = 30): Standard care	Diabetes clinic visits reduced by 88.6%
Carral et al. [38] (Spain)	To assess the effects of a web-based telemedicine system on diabetes clinic visits, monitoring Glu control, maternal, and neonatal outcomes	104 pregnant women, diabetes clinic	(GDM = 77, T1DM = 16, T2DM = 11). Intervention (*N* = 40): Telemedicine and standard care Control (*N* = 64): Standard care	Diabetes clinic visits reduced (3.2 ± 2.3 vs. 5.9 ± 2.3 visits; *p* < 0.001) intervention vs. Control NS difference in maternal outcomes: CS prevalence (30% vs. 40%; *p* = 0.164), MWG (8.4 kg ± 6.5 kg vs. 9.0 kg ± 6.6 kg; *p* = 0.644) intervention vs. control NS difference in neonatal outcomes: LGA prevalence hypoglycaemia (2.5% vs. 3.1%) intervention vs. control
Carolan-Olah and Sayakhot [39] (Australia)	To investigate the effects of an online educational programme on maternal BMI, blood pressure, glycaemic index, and infant birthweight	110 women with GDM, diabetes clinic	Intervention (*N* = 52): Web-based education and standard care Control group (*N* = 58): Standard car	44.2% women in intervention group maintained normal BMI (18.5–24.9 kg/m^2^ post intervention (vs 31%, *p* < 0.001) intervention vs. control Maternal BP * (107/64 mm Hg vs. 109/66 mm Hg), ** (108/68 mm Hg vs. 112/68 mm Hg)] intervention vs. control, NS difference Maternal Glu [(8.8 mmol/L * and 7.3 mmol/L **) vs. (4.9 mmol/L * and 4.7 mmol/L **)] intervention vs. control NBW 2.5 kg−4 kg, NS (92.3% vs. 94.8%) intervention vs. control
Dalfrà et al. [37] (Italy)	To assess the effect of a telemedicine system on maternal and foetal outcome in women with GDM	276 pregnant women attending a diabetes clinic (GDM = 240, T1DM = 36)	Pregnant women with GDM -Intervention (*N* = 88) (Standard care and Telemedicine) -Control (*N* = 115): Standard care Pregnant women with TIDM -Intervention (*N* = 17): Telemedicine and standard care -Control (*N* = 15): Standard care	NS difference in CS and FM (*p* = 0.02)
Guo et al. [34] (China)	To explore the effects of mobile health (mHealth) intervention on pregnancy weight management, blood Glu control, and pregnancy outcomes	124 women with GDM, diabetes clinic	Intervention (*N* = 64): Mobile medical management and standard care Control (*N* = 60): standard care	Significant effect on blood Glu control (4.7 ± 0.2 vs. 5.3 ± 0.3 *p* < 0.001) and MWG, (3.2 ± 0.8 vs. 4.8 ± 0.7, *p* < 0.001) Intervention vs. control NS on pregnancy outcomes: CS (33.3% vs. 25.0%, *p* = 0.325), FM (10% vs. 65.3%, *p* = 0.295) intervention vs. control
Kennelly et al. [31] (Ireland)	To investigate the effect of a smartphone-supported behavioural intervention on the incidence of GDM in overweight and obese women	565 obese women with GDM, diabetes clinic	Intervention (*N* = 278): Smartphone-supported intervention and standard care Control (*N* = 287l): standard care	NS difference in incidence of GDM (15.4% vs. 14.1%, *p* = 0.71) intervention vs. control
Roozbahani et al. [28] (Iran)	To investigate the effects of telephone follow-up on blood glucose level during pregnancy and postpartum screening in women with GDM	80 women with GDM, diabetes clinic	Intervention (*N* = 40): 10 weeks telephone follow-up Control (*N* = 40): 3 weeks telephone follow-up	NS in Glu level at 28 weeks of pregnancy (122.5 ± 19.7 mg/dL vs. 113.2 ± 15.8 mg/dL, *p* = 0.06) intervention vs. control
Miremberg et al. [30] (Israel)	To explore the impact of a smartphone-supported intervention, on patient compliance, glycaemic control, pregnancy outcome, and patient satisfaction	120 newly diagnosed women with GDM, diabetic clinic	Intervention (*N* = 60): Smartphone-supported intervention and standard care Control (N = 60): Standard care	NS difference in LC (84 ± 0.16% vs. 66 ± 0.28%, *p* < 0.001) and Mean Glu (105.1 ± 8.6 mg/dL vs. 112.6 ± 7.4 mg/dL, *p* < 0.001) intervention vs. control,

* Pre-intervention, ** Post-intervention. Abbreviations: BP = Blood pressure, BMI = Body mass index, CS = Caesarean section, FM = Foetal macrosomia, GDM = Gestational diabetes mellitus, Glu = Glucose, Large for gestational age, LC = Level of compliance = Maternal weight gain, NBW = Neonatal birth weight, NS = Not significant.

**Table 4:** Overview of studies reporting on the acceptability of digital tools to support dietary self-management of gestational diabetes mellitus.

Author (Country)	Stated Aim of the Study	Participants, Setting	Study Type-Acceptability Assessment	Key Findings
Given et al. [35] (UK)	To investigate acceptability of using telemedicine in diabetes care of women with GDM	50 pregnant women, diabetes clinic	RCT-user satisfaction, recommendation to others Intervention (*N* = 24): Telemedicine and standard care Control (*N* = 26): Standard care	89% of the participants satisfied and intend to recommend Telemedicine to other women with GDM
Hirst et al. [32] (UK)	To explore women ‘satisfaction with GDM-health system and their attitudes towards their diabetes care	52 pregnant women with GDM, diabetes clinic	Quantitative-user satisfaction, appreciation, recommendation to others	92% of the participants satisfied about using GDM-health system towards diabetes care
Rigla et al. [29] (Spain)	To explore the acceptance of a mobile decision support system for GDM	20 women with GDM	RCT-user satisfaction Intervention (*N* = 20): Mobile technology and standard care Control (*N* = 0)	100% of the participants satisfied to use mobile decision support system for GDM

**Table 5:** Overview of studies reporting on the feasibility of digital tools to support dietary self-management of gestational diabetes mellitus.

Author (Country)	Stated Aim of the Study	Participants, Setting	Study Type	Key Findings
Gianfrancesco et al. [36] (UK)	To explore the feasibility of an online ‘myfood24’ dietary assessment tool in women with GDM	199 women with GDM, diabetes clinic	Mixed method Quantitative (*N* = 216): Questionnaire- actual use, intention to use Qualitative (*N* = 15): Semi-structured interview-perceived appropriateness	‘myfood24′ is feasible (mean 70.9, 95% CI 67.1, 74.6)
Hewage et al. [33] (Singapore)	To investigate perception of patient and health care providers on barriers and preferred intervention to manage GDM.	216 pregnant women with GDM, diabetes clinic	Mixed method Quantitative (*N* = 216): Questionnaire-intention to use, actual use Qualitative (*N* = 15): Semi-structured interview-perceived appropriateness	Web-based support perceived to be feasible in 80.9% of the participants
Sayakhot et al. [27] (Australia)	To explore the feasibility of using a web-based intervention to support on healthy diet and other lifestyle management in women with GDM	116 pregnant women with GDM, diabetes clinic	RCT-Actual use, perceived appropriateness Intervention (*N* = 56): Web-based intervention and standard care Control (*N* = 60): Standard care	Feasible to improve GDM knowledge about GDM (48.2% vs. 46.7%) and high GI carbohydrate (62.5% vs.58.3%)
Skar et al. 2018 [26] (Norway)	To explore the experiences of women with GDM while using pregnancy+ app for health and nutrition information to control blood Glu	17 pregnant women with GDM, 5 diabetic clinics	Qualitative (Semi-structured interview)-perceived appropriateness	The pregnancy+ was perceived to be appropriate in providing easily accessible dietary advice on blood Glu, health, and nutrition in 88.3% of the participants. DA and Glu values in the app not always in agreement with the recommendation from midwives.

## Data Availability

Not applicable.

## Supplementary Materials

The following supporting information can be downloaded at: https://www.mdpi.com/article/10.3390/nu14010010/s1.
