# Supplementary material for: Effectiveness and Usability of Digital Tools to Support Dietary Self-Management of Gestational Diabetes Mellitus: A Systematic Review
Source: Nutrients. 2021 Dec 21;14(1):10. doi: 10.3390/nu14010010 (PMC8746603; doi:10.3390/nu14010010)
Supplement: Supplementary file 1 [file nutrients-14-00010-s001.zip › nutrients-1474464-supplementary.pdf]

Appendix 1: Search strategy

Database searched	Search Terms
**Embase, Cochrane Register of Controlled Trials (CENTRAL), Embase, CINAHL (via EBSCOhost)**	(mobile application OR telehealth/ OR 'mobile phone' OR text messaging OR ('e-health' OR ehealth OR mhealth OR 'm-health' OR telehealth OR smartphone* OR ((mobile OR smart OR Android OR iOS) OR 'm-phone' OR mphone* OR 'cell phone' OR iphone OR ipad OR ipod OR ((app OR apps OR application*) OR portable OR tablet OR Android OR iOS OR web OR online**)**<br>**AND**<br>**(**Gestational diabetes mellitus OR GDM OR gestation* OR gestational diabetes OR diabetes, pregnancy induced OR diabetes mellitus, gestational OR pregnancy, OR pregnancy, hyperglycaemia OR pregnancy glycaemic index OR Pregnancy-Induced Diabetes OR Diabetes, Gestational) OR childbearing* OR maternal* OR maternity OR mother* OR antenatal* OR prenatal* OR perinatal**)**<br>**AND**<br>(diet* OR nutrition* OR food intake OR dietary intake OR food consumption OR calorie intake OR energy intake OR kilojoule intake OR calorie restriction OR diet* therapy OR vitamin OR nutrition* OR dietary intervention**)**<br>**NOT**<br>([Animals])
**Medline (Web of Science), Scopus**	(Mobile Application or Telemedicine or Cell Phones or ("e-health" or ehealth or mhealth or "m-health" OR telehealth) or smartphone* or ((mobile or smart or Android or iOS) ADJ3 (phone* or monitor* or device*)) or iphone or ipad or ((app or (apps or application*) or tablet or Android or iOS or web or online or communication))<br>**AND**<br>(Gestational diabetes mellitus or GDM or gestational diabetes or diabetes, pregnancy induced or diabetes mellitus or gestational or pregnancy, glucose intolerance or pregnancy, hyperglycaemia or pregnancy glycaemic index or Pregnancy-Induced Diabetes)<br>**AND**<br>(diet* or nutrition* or food intake or dietary intake or food consumption or calorie intake or energy intake or kilojoule intake or calorie restriction or diet* therapy or vitamin or nutrition)<br>**NOT**<br>(animal).
